# Nurturing a Society of Learners: Suggestions from Traditional Jewish Pedagogy for Medical Education

**DOI:** 10.5041/RMMJ.10309

**Published:** 2017-07-31

**Authors:** Jacob Urkin, Edward Fram, Allen Jotkowitz, Sody Naimer

**Affiliations:** 1 Moshe Prywes Center for Medical Education and Research, Ben-Gurion University of the Negev, Beer-Sheva, Israel; 2 Siaal Research Center for Family Medicine and Primary Care, Division of Community Health, Faculty of Health Sciences, Ben-Gurion University of the Negev, Beer-Sheva, Israel; 3 Department of Jewish History, Ben-Gurion University of the Negev, Beer-Sheva, Israel; 4 Department of Medicine, Faculty of Health Sciences, Ben-Gurion University of the Negev, Beer-Sheva, Israel

**Keywords:** Educational principles, Jewish tradition, life-long learning, pedagogy, teaching methods

## Abstract

Historically speaking, in many societies a select few carried the burden of preserving and transferring knowledge. While modern society has broadened the scope of education, this is not enough in the medical sciences. We must ensure that all those who pursue a career in medicine become life-long learners who will grow and contribute well beyond their years in medical school. In considering how to attain this goal, we were intrigued by the similarities between generations-old wisdom of teaching and learning methods in Jewish culture and modern educational principles. Both aim to nurture a culture of learners. Our objective was to parallel the methodologies, pedagogic directives, and demands made of students in the Jewish tradition, to the principles used in medical education today. We surveyed the traditional Jewish culture of teaching and learning. We compared it to modern medical teaching methods and looked to see what lessons might be gleaned. In the traditional Jewish community, life is focused on education, and producing “learners” is the ideal. This culture of learning was developed over the generations and many educational methods are similar to modern ones. Some of the pedagogic principles developed successfully in Jewish society should be considered for adaptation in medical education. Further comparative research could help to expand the ways in which we teach medicine.

## INTRODUCTION

Teaching and learning are embedded in human development. Children start intuitively by processing sensory inputs that are enforced by parental reciprocal trajectories with their offspring. Every human society has both structured and unstructured means of educating their youth to fulfill their role in life as adults. Jewish culture has a reputation of cherishing “learning” and transferring this passion from one generation to the next.^1^ Its perceived successes justify delving deeper into the intricacies of this system to uncover some of the methods that characterize it. The purpose of this paper is to summarize some traditional Jewish methods of teaching and learning and relate them to modern educational principles which also apply to medical education.

## BACKGROUND

Jewish culture is based on a translation of biblical rules and principles into practice. Throughout the past 2000 years, if not longer, Jewish scholars have debated how best to accomplish this, and records of their deliberations and conclusions have created the classical “Jewish library.” Beyond the Hebrew Bible, the Talmud, a compilation of rabbinic thought from the first to seventh centuries, and hosts of commentaries that it garnered through the ages have become the foundation texts. There are also legal treatises that explain, elaborate, and help adjust the laws to daily life. Together with communal customs, these texts present the traditions that every observant Jew is expected to master and practice. These objectives constitute a significant learning burden on each individual and necessitate perseverance and efficient learning techniques.^2^

## SETTING THE STAGE FOR TOTAL IMMERSION

The cultural centrality of study is demonstrated in the home environment. Here books often enjoy a place of prominence.^3^ Ideally, children witness parents and siblings who engage in study on a daily basis, and the young are taught to value and emulate this. The written word and mimetic religious culture dictate every aspect of life.^4^

According to the ideals of this community, preoccupation with learning is almost an obsession, and time is an extremely valuable resource. Even social and religious events involve group learning. For example, a meal without a word of learning is considered as if one ate from “the sacrifices of the dead” (Avot 3.3).^5^ No individual is exempt from the obligation to study, each according to his level of skill. In many contemporary communities, the emphasis on learning now extends to women as well. Any activity that detracts from learning must be justified. Going to work, or even visiting the doctor, serves the principal objective and leads to continued, or even better, learning thereafter.

Mindfulness of learning is a constant state of consciousness, and reminders to pursue study at all times are everywhere. The central dogma of Judaism is recited twice daily as part of the prayer service. The *Shema Yisrael* (“Hear Israel” [Deut. 6.4]) prayer not only expresses complete devotion to a monotheistic God but formally commands that one teach the young morning and night, whether at home or on the road (6.7) (“*ve-shinantam le-banecha*” [you shall reiterate them to your offspring]). Not surprisingly, orthodox Jews often carry a book or other learning material wherever they go, and studying while waiting in line or travelling is common.^6^ Writings on parchment placed in a small capsule on every door and gate (6.9) (the so-called “*mezuza*” placed on the doorpost) reinforce this message.

On the Sabbath, exempt from routine materialistic preoccupations, one is expected to engage in study. Holy days are characterized by preparations that include the learning of related laws and customs, and education is an intrinsic component of the holy days. A detailed description of the pedagogic methods and principles used at the Passover Seder has been discussed by Glick.^7^

Children enter a more formal setting for learning at an early age. On their first day in “*Cheder*,” the Jewish toddlers’ classroom, children eat cookies shaped as Hebrew letters dipped in honey to form a connection in the mind of the young between sweetness and pleasure and study.^8^ The rabbinic leadership recognized the importance of continuing education and developed the notion of studying a page of the Talmud each day (“*daf yomi*”) which has become part of the routine for many laypeople.

This sort of environment in which every (male) member of the community is expected to devote each free waking moment to study stands in contrast to other cultures that have similar approaches but only for select scholars and religious devotees (monks, for example). Not surprisingly, literacy rates in Jewish communities have generally been higher than those among other groups among whom Jews lived.^9^

Current educational systems often utilize total immersion for short periods, such as crash courses for the purpose of acquisition of a new language, to enhance learning. Such experiences are costly and limited in time and scope. At the opposite extreme, the Jewish system strives to create environments that stimulate learning through all phases of growth and development from birth to death. This is an experience-based education, which for the most part is free of charge. Moreover, the classical study hall (“*beit midrash*”), which is generally endowed with a significant library, is open to the public 24/7, and there is continual formal and/or informal instruction there.

## PROVERBS THAT CAPTURE THE WISDOM OF TEACHING AND LEARNING

There are many axioms in classical Jewish literature that relate to study and may prove helpful in identifying some of the values in Jewish education that might be considered in modern medical education (Table 1).

**Table 1:** Talmudic Maxims that Express Esteem for Learning and their Educational Implications.

Jewish Proverbs^*^	Educational Principle
I have learned from all my teachers [Psalms 119.99 with Avot 4.1]	One must be willing to learn from everyone; do not miss a learning opportunity
Sit in dust at the feet of Sages and drink their words thirstily [Avot 1.4]	Seek out and strive to learn as much as you can from scholars
Find yourself a teacher and acquire a fellow student [Avot 1.6]	Responsibility for study is the student’s; look for a mentor and study in tandem with a friend (small-group learning)
It is one’s duty to hear the preaching of scholars [Babylonian Talmud (BT), Yevamot 20a]	Strive to attend the teaching of prominent teachers
A very strict teacher cannot teach [Avot 2.5]	Teaching demands flexibility
Educate a child according to its abilities [Proverbs 6.6]	Student-centered teaching
One who is embarrassed easily is not able to learn [Avot 2.5]	Encourage all students, even the meek, to participate and ask questions
May the honor of your student be as precious as your own honor [Avot 4.12]	Respect the students
If you see a pupil whose study is laborious, his study is badly arranged [BT, Ta’anit 7a,b]	A well-considered curriculum eases the learning process; a teacher must follow the progress of students and, if necessary, make adjustments to meet their needs
I have learned much from my teachers, more from my colleagues, and the most from my students [Ta’anit 7a]	Preparing for a lecture augments one’s own learning, but learning from the questions of one’s students can be even more enlightening
Make regular time for learning Torah (the law) [BT, Shabbat 31a]	Set a fixed time for your learning
If you forsake study for one day, it will forsake you for two [Jerusalem Talmud, Berachot 14d]	Learn every day; become a life-long learner (continuing medical education)
Do not say, “when I have time I will study,” lest you not have time [Avot 2.4]	Make learning a priority that is not deferred
One who studies his lesson a hundred times is not the same as one who studies it one hundred and one times [BT, Hagigah 9b]	Repeated learning over and over again is beneficial and well advised even when seemingly superfluous for it deepens knowledge and understanding
It is not incumbent on you to complete the whole task, but you cannot neglect it [Avot 2.16]	A good student (and doctor!) never gives up
Any talmudic scholar whose inside is not like his outside is not a talmudic scholar [BT, Yoma 72b]	Educational and professional integrity
Every place of study has its own innovation [BT, Hagigah 3a]	New ideas are generated in every academic environment
Who is a scholar? One who can quote the law at any time and place [BT, Shabbat 114a]	Become an expert in what you do
The wits of a scholar are sharpened by his fellow scholars [Bereshit Rabba] As iron whets another, so scholars sharpen each other’s wits [BT, Ta’anit 7a]	Discussion and debate ensure deeper understanding and learning
Study leads to action [BT, Kiddushin 40b]	Theory precedes and modifies practice

* References to sources are in brackets.

## CLIMBING THE SOCIAL LADDER BY SCHOLAR MERIT

As learning and living according to the law are the main purposes of life, they require one to give up on other activities. The reward for this is personal satisfaction and prestige in this world and the promise of reward in the next. The titles bestowed on outstanding students and scholars exemplify this value system. In elementary school, a student who attends off-hours classes is called a “*matmid*” [a perpetual learner], emphasizing perseverance in study. The term “*talmid chacham*” [a very wise student] refers to those who excel at an advanced level—and even to the greatest scholars—and assumes constant study. One who makes new connections between ideas and generates new hypotheses is called “*mechadesh chiddushim*” [an innovator of new ideas]. Two special terms adorn those with the greatest of talents: “*tochen harim*” [one who grinds mountains against each other] is reserved for scholars who can integrate and synthesize ideas, and “*Sinai*” (as in the sacred mountain) is a moniker reserved for scholars with complete control of the sources, well versed in all texts, and able to quote from any part of the rabbinic corpus at will.

In this value system, knowledge of Jewish culture is held in the highest esteem and is a means to status that has practical ramifications, particularly in the realm of matchmaking. Arranged marriages are the rule in ultra-orthodox society, and one of the most important attributes of a potential groom is his credentials as a learner. The best scholars and their families are the nobilities of the orthodox society. Similarly, the credentials of a potential bride often depend on the academic prestige of her father and brothers. In some ways this resembles trends in secular education systems where outstanding students are given prizes based on scholarly merit, a common way of motivating students to excel.

## TEACHING METHODS AND SUPPORT TO LEARNERS

With study of paramount importance, educational techniques have been honed in Jewish communities.^10^ That these systems have thrived through the centuries is a testimony to their effectiveness. Table 2 describes a number of these methods and correlates them with modern educational principles.

**Table 2:** Traditional Jewish Means of Education and their Modern Parallels.

Traditional Jewish^*^	Modern Teaching
Learning is motivated internally (by faith) and externally (by social pressure)	Learning is motivated internally (through idealism, curiosity) and externally (professional demands)
Consideration of the student’s needs and abilities in pacing study	Student-centered teaching; personal learning plan and portfolio^11^
Arguments based on citations	Referenced arguments (evidence-based medicine)
Learning how to think independently and substantiate opinions (“*ra'ayah*”)	Evidence-based decision-making skills
Use of biblical narratives as basis for legal discussions (e.g. 1 Sam. 1 with Berachot 31b)	Learning by using narrative, literature, arts, media, etc.
Rhetorical method^12^	Challenge students by asking questions; transform all students into active learners
Encourage students to ask questions, even if theoretical^12^	Encourage students to ask questions
Encourage the examination of problems from different perspectives; force students to articulate and defend their positions before teachers and peers in an open debate^12^	Encourage different opinions and lead a debate
Introduce multi-valence readings of texts through the use of commentaries from different times and places	Explore more than one interpretation for an observation
Recognition of different layers of text, “*Pshat*” (literal), “*Remez*” (allegory), “*Drash*” (metaphorical), “*Sod*” (mystical)	Generate deeper learning by acquiring more detailed data or new theories; the use of imagination
Learning in partnership with a fellow student (“*Hevruta*;” a pair that may learn together for many years)^13^	Peer teaching and learning in small groups
Group review of texts to allow weak students to learn from stronger ones	Group learning among students including via social networks
The junior member(s) of a rabbinical tribunal have to express opinions before others	On clinical rounds, students express opinions before more senior team members, encouraging openness and unbiased ideas
Preparing sources before the actual group discussion (“*Habura*”)	The flipped classroom
Encourage repetition and memorization in order to know, cite, and build analogies	Immediate recall of sources as a basis for decision-making
“*Notricon*” use of acronyms and rhymes to support recollection of learning material	Use of acronyms, acrostics, and rhymes to support recollection of learning material
Dividing the public reading of the Pentateuch in the synagogue into an annual cycle reinforces knowledge of the foundation text	Spiral teaching that strengthens learning at each cycle; division of teaching material throughout planned scholastic year
Try to resolve textual conundrums through commentaries^12^	Problem-based learning;^15^ case-based learning
Connect daily events to the current learning material^14^	Motivation by clinical relevance and actual cases
Law is summarized so that there is practical guidance (e.g. “*Shulchan aruch*” and “*Mishnah Berurah*”)	Summary of principles, decision trees, algorithms
The student is expected to devote time to ethics, biblical studies, practical law, etc. independently of formal curriculum	Self-directed learning portfolio
Learning the laws associated with a holy day before its advent	Theory is learned before practice
Rehearsal before practical performance (e.g. children perform precepts before they are actually obligated to do so)	Simulation before practice
Cross-textual learning that connects related topics (“*Sugyot*”) and concepts to “the big picture”	Cognitive map
“*Din ve-heshbon*”—students are expected to self-examine their behavior	Mindfulness, reflection (meta-cognition), accountability
“*Bein Hazmanim*”—set vacations to allow rejuvenation	Protected time to allow teachers and students alike to rest from professional demands
“*Shiur*”—a lecture to a large group usually by a rabbi	Large-group teaching by a distinguished professor
“*Hameshiv*”—an individual who provides one-on-one instruction and fulfills the position of a knowledgeable mentor	Private teacher; mentor
“*Hamashgiach*”—a person who provides ethical and spiritual guidance as well as individual consultation on non-academic issues	A counselor, group leader, or a mentor
“*Moreh Shiur*”—teaching to a large group by someone who is not a rabbi	Large-group teaching by tutors
“*Meshamesh*”—learning skills through apprenticeship	Apprenticeship, internship
“*Shimush Chachamim*”—a young student is assigned to follow a senior student and learns from him the code of behavior	Mentoring, role modeling, professionalism
Tests before being promoted to be a Rabbi or a “*Dayan*” (a judge in a rabbinic court)	Competency-based exams in order to achieve licensing (e.g. medicine)

* Hebrew terms are in italics and enclosed in quotation marks.

## POSSIBILITIES FOR TRANSFER OF SKILLS

From an early age, Jewish children are invariably exposed to more than one language. The language spoken at home is often that of the host country, but in many homes a “Jewish language” is spoken, the most common being Yiddish, which evolved from German, or Ladino, from Spanish. In such environments, children will eventually have to learn the language of the host culture as well. In addition, there is a demand that children learn biblical Hebrew and eventually master Aramaic, the language of the Talmud.^12^ There is also a need to learn a number of different character sets (i.e. Hebrew, Latin, and special fonts used for the printing of various texts). Although this is daunting, most children master the challenge. The brain of the young is flexible and adaptable to learn more than one language at the same time. There is scientific support to claim that learning more than one language in the early years may have an advantage in the intellectual development.^15^

The traditional curriculum includes an enormous amount of text study. Little effort is devoted to sciences, arts, or vocational knowledge. Exposure to the media is limited and controlled. Television or free roaming on the internet is prohibited. Surprisingly, these limitations often—but not always—do not pose a significant barrier for those who pursue a career in secular higher education. Many who were educated under traditional guidance succeed in closing the gaps of knowledge in relatively short order and are accepted to universities. It seems that the abilities developed through the use of traditional methods help in absorbing unfamiliar material in a short period. Excellence in learning, sophisticated thinking processes, and innovation characterize many of these individuals in the sciences and humanities.^16^

## SUMMARY

The Jewish educational principles that contribute to the success in raising outstanding scholars can be summarized by the following standards:

1. Glorification of scholars and learning.
2. Development of a variety of teaching methods and opportunities for learning.
3. Flexibility in addressing students’ needs.
4. Addressing the relevance of theoretical learning to daily life.
5. Prioritization of values.
6. Encouragement of curiosity and questioning as a generator of solutions.
7. Becoming an expert on minute details while maintaining awareness of the big picture.
8. Being respectful to teachers, peers, and students.
9. Maintaining the habits of life-long learning as an obligation.
10. Self-awareness and accountability.

These principles fit the objectives of medical education, to which they must add their own unique aspects. Communication skills, sociocultural awareness, professionalism, patient privacy, and dealing with death are on the list. The above standards can serve as a platform for planning the medical curriculum and its implementation.

Jewish tradition is but one example of a culture that glorifies scholarship. From the traditional Jewish perspective, many of the new methods of teaching and learning are no more than old wine in new vessels. Scientific research on the methodology of the Jewish educational experience is still limited. Here we have presented a “taste” of this vast system by describing some of its goals and methods. The ideal of life-long learning is shared both by the Jewish tradition and the field of medicine. Given the success of Jewish tradition in achieving this goal, more should be done to learn from its educational models—as well as those of other cultures—and consider their potential applications to medical education.^17^
